# Synergistic Nitrogen-Doping and Defect Engineering in Hard Carbon: Unlocking Ultrahigh Rate Capability and Long-Cycling Stability for Sodium-Ion Battery Anodes

**DOI:** 10.3390/ma18102397

**Published:** 2025-05-21

**Authors:** Na Li, Hongpeng Li, Haibo Huang

**Affiliations:** 1 Key Laboratory of Bio-Based Material Science and Technology (Ministry of Education), Northeast Forestry University, Harbin 150040, China; 2 School of Automotive Engineering, Nantong Institute of Technology, Nantong 226001, China; 3 College of Mechanical Engineering, Yangzhou University, Yangzhou 225127, China

**Keywords:** sodium-ion battery, anode, hard carbon, N-doped, defects

## Abstract

Hard carbon (HC) anodes for sodium-ion batteries (SIBs) face challenges such as sluggish Na⁺ diffusion kinetics and structural instability. Herein, we propose a synergistic nitrogen-doping and defect-engineering strategy to unlock ultrahigh-rate capability and long-term cyclability in biomass-derived hard carbon. A scalable synthesis route is developed via hydrothermal carbonization of corn stalk, followed by controlled pyrolysis with urea, achieving uniform nitrogen incorporation into the carbon matrix. Comprehensive characterization reveals that nitrogen doping introduces tailored defects, expands interlayer spacing, and optimizes surface pseudocapacitance. The resultant N-doped hard carbon (NC-2) delivers a remarkable reversible capacity of 259 mAh g^−1^ at 0.1 A g^−1^ with 91% retention after 100 cycles. And analysis demonstrates a dual Na⁺ storage mechanism combining surface-driven pseudocapacitive adsorption (89% contribution at 1.0 mV s^−1^) and diffusion-controlled intercalation facilitated by reduced charge transfer resistance (56.9 Ω) and enhanced ionic pathways. Notably, NC-2 exhibits exceptional rate performance (124.0 mAh g^−1^ at 1.0 A g^−1^) and sustains 95% capacity retention over 500 cycles at 1.0 A g^−1^. This work establishes a universal defect-engineering paradigm for carbonaceous materials, offering fundamental insights into structure–property correlations and paving the way for sustainable, high-performance SIB anodes.

## 1. Introduction

With the rapid increase in global demand for large-scale energy storage, sodium-ion batteries (SIBs) have become an important supplementary technology to lithium-ion batteries. This is primarily attributed to the abundant sodium resources on Earth and the similar working principle to lithium-ion batteries [1,2,3,4]. However, the larger ionic radius of Na^+^(1.02 Å compared to 0.76 Å for Li⁺) severely restricts the sodium storage performance of traditional graphite anodes, with capacities typically less than 35 mAh g^−1^. This limitation has spurred extensive research efforts to develop novel carbon-based anode materials for SIBs [1,5,6,7,8].

In the system of SIBs, the performance of anode materials plays a decisive role in determining the overall battery performance. Carbon-based materials have become a research hotspot for SIB anode materials, owing to their abundant resources, low cost, excellent electrical conductivity, and excellent cycling stability [9,10]. Among them, hard carbon materials are considered one of the most promising anode materials for SIBs. They possess a unique turbostratic graphite structure, larger interlayer spacing, and a rich pore structure, which enable them to accommodate the insertion and extraction of sodium ions effectively [11]. As a result, hard carbon materials exhibit a relatively high theoretical specific capacity and a low working potential [12]. Nevertheless, pristine hard carbon materials still suffer from several drawbacks, such as low initial Coulombic efficiency and limited rate performance, which hinder their practical applications [13,14,15]. For instance, Jin et al. prepared a N/P/O co-doped biomass peanut shell hard carbon anode material, with an initial Coulombic efficiency of 29.6% at a current density of 0.1 A g^−1^ [16]. Qin et al. prepared a carbon sheet doped with both nitrogen and phosphorus as the anode material for sodium-ion batteries. At a current density of 0.1 A g^−1^, it exhibited an initial coulombic efficiency of 53.1% [17]. The main reason for the low initial Coulombic efficiency was the formation of a SEI film on the electrode surface, which led to the decomposition of some electrolytes and resulted in a significant irreversible capacity.

Hard carbon (HC) materials, characterized by their disordered graphite-like microcrystalline structures and abundant nanopores, demonstrate excellent sodium storage capabilities, with capacities ranging from 200 to 300 mAh g^−1^ [18,19,20,21]. Recent studies have indicated that heteroatom doping, including nitrogen (N) [22], sulfur (S) [23,24], and phosphorus (P) [25,26,27], can effectively modulate the electronic structure and interlayer chemical environment of hard carbon [28]. Among these, nitrogen doping has attracted significant attention due to its unique electron-donating properties, enhancement of surface pseudocapacitance, and its ability to regulate the electron cloud distribution of carbon materials. Given nitrogen’s relatively high electronegativity, the chemical bonds formed between nitrogen and carbon possess a certain polarity, which can effectively adjust the electron cloud distribution of carbon materials, enhance charge transfer capabilities, and thereby improve the electrical conductivity of the materials [29,30]. In nitrogen-doped hard carbon, the distinct nitrogen species, such as pyridinic, graphitic, and pyrrolic, play pivotal roles in enhancing sodium storage performance through unique structural and electronic interactions. Pyridinic nitrogen, situated at carbon layer edges or defects, exhibits high electronegativity due to its lone pair electrons, enabling strong electrostatic adsorption of sodium ions and facilitating rapid diffusion via low-energy barrier channels. Its presence also introduces edge defects that serve as additional storage sites. Graphitic nitrogen, embedded within the carbon lattice, enhances electronic conductivity by increasing carrier concentration and expands the carbon layers, thereby reducing sodium-ion insertion resistance and forming stable closed pores that accommodate quasi-metallic sodium clusters. Pyrrolic nitrogen, residing in five-membered rings, balances reversibility and capacity by moderating sodium-ion binding, contributes pseudocapacitance through redox-active groups, and improves structural flexibility to mitigate volume changes during cycling. Collectively, these nitrogen species induce lattice distortions and defects, synergistically optimizing the material’s sodium storage capabilities through multi-scale structural tuning. Theoretical calculations have shown that nitrogen-doped sites can reduce the Na⁺ adsorption energy to the range of −1.5 to −2.0 eV, significantly improving the reaction kinetics [31,32]. Moreover, traditional doping methods often lead to excessive defect formation in the carbon skeleton, which undermines the cycling stability of the materials. Nitrogen atom doping can significantly enhance the electronic conductivity and surface wettability of hard carbon while introducing pseudocapacitive active sites [33,34]. This doping method helps optimize the surface chemical properties of hard carbon materials, promotes the formation of a stable solid electrolyte interphase (SEI) film, and thereby improves the cycling stability of batteries [35,36]. Among various nitrogen sources, urea (CH_4_N_2_O) is regarded as an ideal choice for nitrogen doping due to its extremely high nitrogen content of 46.6%. During the hydrothermal carbonization process, urea can form a hierarchical porous framework and retain abundant oxygen-containing functional groups, providing ideal anchoring sites for subsequent nitrogen doping. The free radicals introduced by nitrogen doping facilitate the highly reversible adsorption of sodium ions. Incorporating heteroatoms into the carbon framework can increase the interlayer spacing, reserving sufficient space for the insertion and accommodation of sodium ions [37,38,39,40].

Herein, we report a novel strategy of synergistic nitrogen doping and defect engineering in hard carbon for SIB anodes. By precisely controlling the nitrogen-doping process and introducing well-defined defects, we aim to achieve a remarkable improvement in both the rate capability (0.1 A g^−1^ and 244.9 mAh g^−1^) and the long-cycle stability of hard-carbon anodes. This chemical bonding strategy enables the doping of nitrogen elements and increases the specific surface area. The optimized anode exhibits a reversible capacity of 259 mAh g^−1^ at a current density of 0.1 A g^−1^, with an initial Coulombic efficiency of 65.7%, and shows excellent rate performance. Crucially, at a current density of 0.1 A g^−1^, after 100 cycles, its capacity can still be maintained at 91%, outperforming the undoped biomass-derived carbon materials. The innovative aspect of this work lies in the simultaneous optimization of nitrogen-doping and defect-engineering techniques, which enables a synergetic effect that has not been fully explored before. This approach provides new insights into the fundamental understanding of sodium-ion storage mechanisms in hard carbon materials and paves the way for developing high-performance SIB anodes for practical applications.

## 2. Results and Discussion

### 2.1. Structural Evolution and Chemical Modulation of N-Doped Carbon (NC)

**Scheme 1:**
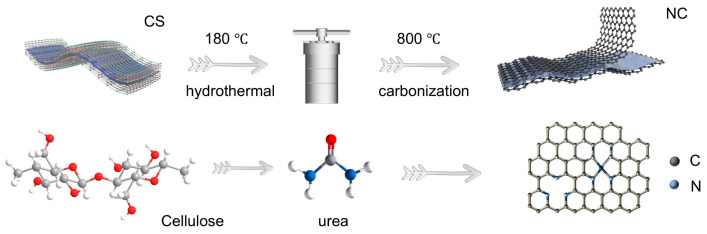
Schematic illustration of the synthesis process for water-thermal-assisted nitrogen-doped hard carbon materials.

Scheme 1 delineates a scalable synthesis protocol for nitrogen-doped corn stalk-derived hard carbon, employing biomass-derived corn straw (CS) as the carbon precursor and urea as the nitrogen source. The process initiates hydrothermal treatment to facilitate cross-linking and the polymerization of molecular precursors, forming an N-enriched intermediate. Subsequent pyrolysis under an argon atmosphere at elevated temperatures drives simultaneous carbonization and nitrogen incorporation into the carbon framework, generating defect-rich NC materials. Systematic experiments were performed to optimize the urea-to-CS mass ratio, establishing a critical balance between nitrogen-doping efficacy and structural integrity. For comparative analysis, pristine HC was synthesized under identical conditions without nitrogen doping, enabling direct evaluation of nitrogen-induced structural modifications (e.g., expanded interlayer spacing and defect density) and their electrochemical consequences. This methodology not only ensures controlled nitrogen distribution within the carbon matrix but also highlights the pivotal role of defect engineering in enhancing sodium-ion storage kinetics and cycling stability.

**Figure 1:**
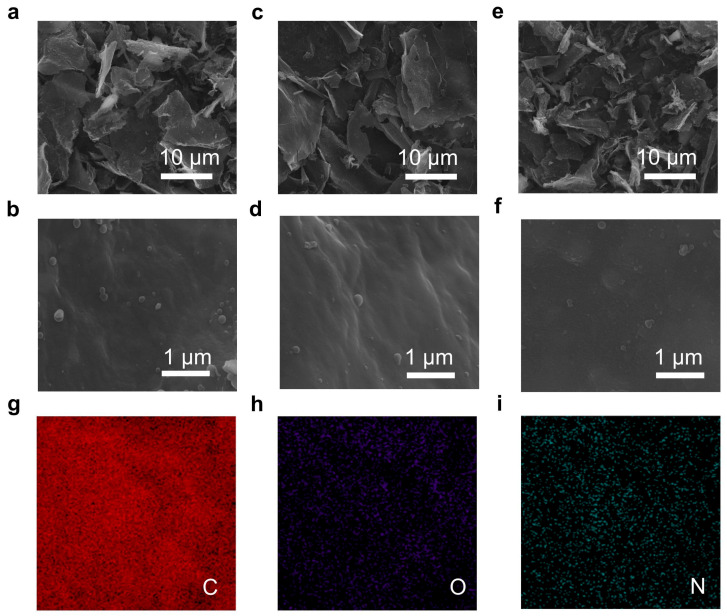
Scanning electron microscopy (SEM) images of (**a**,**b**) NC-1, (**c**,**d**) NC-2, (**e**,**f**) NC-3. (**g**–**i**) Elemental mapping of NC-2.

Upon examining the microscopic morphology of NC via SEM images (Figure 1a–f), it was observed that all samples exhibited a lamellar or layered structure. Nevertheless, variations were noted in terms of size and degree of agglomeration. The flaky structure of NC-1 is relatively large and shows a certain degree of agglomeration, while the structure of NC-3 is more fragmented with relatively smaller lamellar sizes. This morphological disparity is related to the nitrogen-doping content, as different nitrogen contents can influence the growth and stacking patterns of the material during synthesis. Upon high-magnification SEM observation, the surface of NC-1 has numerous wrinkles, which are caused by nitrogen doping. The surface of NC-3, on the other hand, features some tiny pores and wrinkles. The micro-morphological structure of the surface can affect the contact area between the material and the electrolyte, as well as the adsorption and diffusion of ions. Compared with NC-1 and NC-3, the flaky structure of NC-2 is more evenly distributed, with less agglomeration. Under high-magnification SEM, the surface of NC-2 is relatively smooth and flat, with some wrinkles present. Energy dispersive spectrometer (EDS) elemental scanning (Figure 1g–i) confirms the presence of C, O, and N elements. Meanwhile, it demonstrates that the C, O, and N elements are uniformly distributed on the carbon matrix. This can fully improve the structure of the carbon material, which is more conducive to the enhancement of electrochemical performance. Uniform nitrogen doping is beneficial for forming stable active sites within the material, altering the electronic structure of the material, and thus enhancing its electrochemical performance.

**Figure 2:**
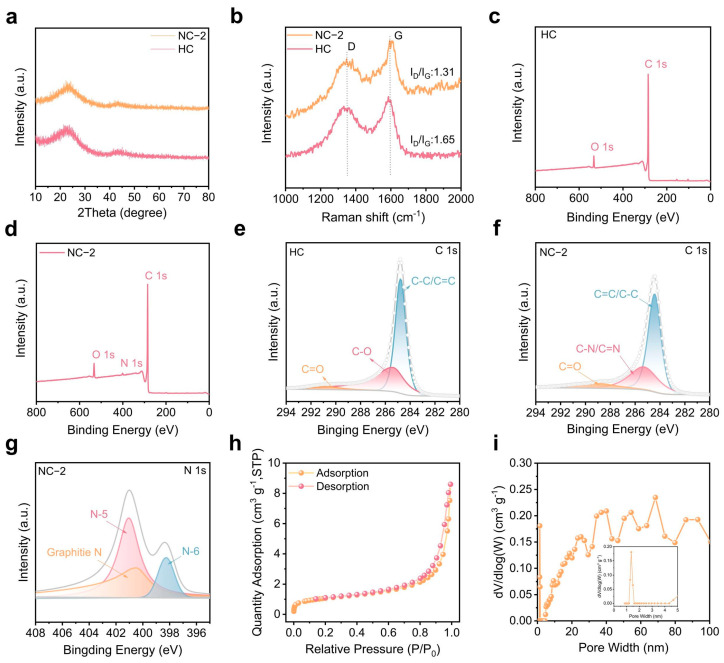
(**a**) XRD patterns, (**b**) Raman spectra, (**c**,**d**) XPS curves, (**e**,**f**) C 1 s partial peak fitting of HC and NC-2, (**g**) N 1 s partial peak fitting of NC-2, (**h**,**i**) N_2_ adsorption/desorption isotherms of NC-2. Inset: pore size distributions.

The crystal structures of the carbon materials were characterized by X-Ray Diffraction (XRD) (Figure 2a and Figure S1a). Two diffraction peaks were observed at 24° and 43°, corresponding to the (002) and (100) planes of graphitic carbon, respectively [41]. The weak and broad characteristics of the diffraction peaks indicate the amorphous nature of these carbon materials. Compared with HC (0.37 nm), NC-2 has a lower (002) diffraction peak and larger d_002_ interlayer spacing (0.38 nm). The larger interlayer spacing is beneficial for reducing the diffusion barrier of Na⁺ between the carbon layers. The Raman spectra of NC and HC (Figure 2b and Figure S1b) show characteristic peaks of carbon materials at 1350 cm^−1^ (D-band) and 1590 cm^−1^ (G-band) [42]. The D-band represents defects and disordered structures in carbon materials, while the G-band represents the degree of graphitization. A larger I_D_/I_G_ ratio indicates more defects in the material. The I_D_/I_G_ ratios of NC-1, NC-2, and NC-3 are 1.28, 1.31, and 1.33, respectively, indicating that as the nitrogen-doping content increases, the number of defects in the samples increases, and the degree of graphitization is higher than that of HC (1.65).

X-Ray Photoelectron Spectroscopy (XPS) was employed to determine the chemical composition and state of the NC materials (Figure 2c–g). In the full XPS spectra of all materials, two distinct peaks were observed near 285 eV and 538 eV (Figure 2c,d), corresponding to C 1s and O 1s, respectively. For the NC materials, an additional N 1s peak emerged, which verifies that N elements were successfully doped into the carbon matrix under high-temperature carbonization conditions. Subsequently, peak-fitting was performed on the samples. In the C 1s spectra, C-N/C=N characteristic peaks, different from those in HC materials, were observed, indicating that the introduction of nitrogen atoms leads to the redistribution of electron charge on the carbon materials [43]. This generates more active sites, thereby enhancing the interaction with reactant molecules and promoting more efficient chemical processes (Figure 2e,f). In the O 1s spectra, three peaks corresponding to C=O, C-O, and COOH were present (Figure S2), confirming the presence of numerous oxygen-containing functional groups in the materials. These oxygen-containing functional groups can adsorb more Na⁺. Additionally, they contribute to the formation of a large number of defect structures, expanding the interlayer spacing of carbon layers and improving the surface wettability of the electrode. In the N 1s spectra, the types of nitrogen doping corresponded to pyridinic nitrogen, pyrrolic nitrogen, and graphitic nitrogen [44]. The presence of pyridinic nitrogen and pyrrolic nitrogen introduces a large number of defects and active sites, which is beneficial for enhancing charge transfer and promoting the surface pseudocapacitive effect (Figure 2g). Among them, the proportions of pyrrole nitrogen, pyridine nitrogen, and graphite nitrogen are 40.4%, 29.1%, and 30.5%, respectively. Furthermore, Brunauer–Emmett–Teller (BET) analysis and the pore size distribution of NC-2 material were conducted (Figure 2h,i). The isotherm exhibited a Type IV pattern, indicating that the material is predominantly mesoporous [45]. The BET analysis reveals that the specific surface area of NC-2 is 80.0 m^2^ g^−1^. Meanwhile, the pore size distribution of NC-2 showed that the material has a rich pore structure (macropores, mesopores, and micropores), which can facilitate the full infiltration of the electrolyte into the electrode material, improve electrical conductivity, and enable the rapid diffusion of Na^+^.

### 2.2. Electrochemical Performance of the NC Anode of SIBs

**Figure 3:**
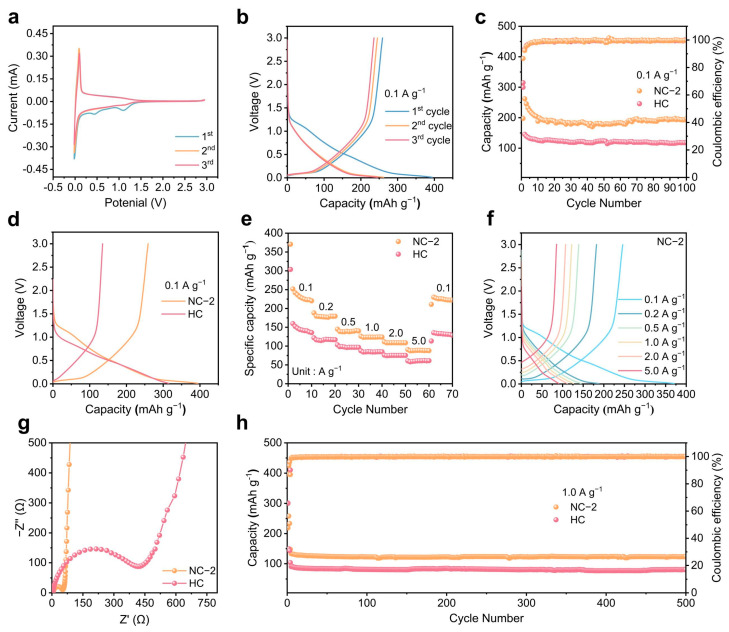
(**a**) CV curve of NC-2 at a sweep rate of 0.1 mV s^−1^. (**b**) GCD profile of NC-2 at a current density of 0.1 A g^−1^ for the first three cycles. (**c**) Cyclic stability of NC-2 and HC samples at 0.1 A g^−1^. (**d**) GCD comparison curves of NC-2 and HC at 0.1 A g^−1^. (**e**) Rate performance of NC-2 and HC. (**f**) The GCD curves of NC-2 under different current densities. (**g**) Electrochemical Impedance Spectroscopy (EIS) Nyquist plots of NC-2 and HC, revealing their charge transfer resistance and ion diffusion kinetics. (**h**) Cycling stability of NC-2 and HC at a higher current density of 1.0 A g^−1^.

The materials were assembled into CR2032-type sodium-ion half-cells, and electrochemical tests were carried out to determine the optimal urea content ratio. In the CV curves (Figure 3a), a pair of redox peaks was observed at 0.1 V, indicating the insertion/extraction of sodium ions in the electrode materials. A reduction peak emerged at 1.25 V, signifying the insertion of sodium ions. When compared with the first cycle CV curve of HC (Figure S3), NC-2 exhibited a higher peak current, indicating that NC-2 has faster reaction kinetics. Nitrogen doping optimized the electronic and pore structures of the material, facilitating the contact and reaction between ions in the electrolyte and the electrode material and accelerating the charge transfer process. This makes it more conducive to the rapid progress of electrochemical reactions compared to HC. Subsequently, the high overlap of the second and third cycles of the CV curves indicates that the storage of sodium ions in NC-2 is highly reversible and remains stable during cycling. In the CV curve, the current changes corresponding to the redox peaks are caused by the Faraday reaction, that is, a chemical reaction of electron transfer, which is the intercalation and deintercalation reactions of sodium ions. The magnitude of the peak current reflects the number of sodium ions involved in the reaction and the reaction rate. The larger the peak current, the more charges are involved in the Faraday reaction at that potential; that is, more sodium ions undergo the intercalation/deintercalation process. Apart from the current corresponding to the redox peak, the background current is mainly caused by non-Faraday processes. These may include capacitive charging on the electrode surface and adsorption/desorption processes (such as chemical adsorption without electron transfer, etc.). Although non-Faraday processes do not directly participate in the intercalation/deintercalation reactions of sodium ions, they can affect the overall electrochemical behavior of the electrode, such as influencing its charging and discharging efficiency and kinetic processes. In summary, nitrogen doping enhances the electrochemical performance of the material by increasing defects and active sites. For the Galvanostatic Charge–Discharge (GCD) curves of NC anode materials at a current density of 0.1 A g^−1^ (Figure 3b and Figure S4), it was observed that the GCD curves of all materials had a similar shape, with a sloped region and a plateau region, and were dominated by the slope, representing the insertion and extraction of sodium ions and the adsorption and desorption at active sites, respectively. In the GCD curve, except for the plateau period, there are non-Faraday process influences during the stage of rapid voltage rise or fall. During the charge and discharge plateau period, a typical Faraday reaction occurs, namely, the intercalation and deintercalation of sodium ions. The amount of charge passing through is directly proportional to the number of sodium ions involved in the reaction. According to Coulomb’s law, the corresponding capacity can be calculated. The capacity contribution during the plateau period mainly comes from the Faraday reaction, which is the main manifestation of the sodium storage capacity of the material. The initial discharge/charge capacities of NC-2 at 0.1 A g^−1^ were 259/394.4 mAh g^−1^, and the Coulombic efficiency (ICE) was 65.7%. The lower initial efficiency is due to the irreversible reaction with the surface functional groups or the formation of a solid electrolyte interface (SEI). In the long-term cycling stability test at 0.1 A g^−1^ (Figure 3c), it was found that NC-2 could still retain 91% of its initial capacity (197 mAh g^−1^) after 100 cycles, significantly higher than that of the HC material (118.2 mAh g^−1^). By measuring and comparing the first cycle initial charge–discharge curves of the two samples at a current density of 0.1 A g^−1^ (Figure 3d), the initial specific capacity and ICE of NC-2 were significantly higher than those of HC (42.95%), which directly demonstrates the enhancement of the sodium storage capacity of the material by nitrogen doping. Meanwhile, NC-2 showed a more stable and appropriate voltage plateau.

The rate capability of NC-2 was systematically evaluated across current densities spanning 0.1 to 5.0 A g^−1^ (Figure 3e and Figure S5). The material delivered high specific capacities of 244.9, 179.4, 139.8, 124.0, 109.0, and 88.4 mAh g^−1^, respectively, showcasing minimal capacity degradation under aggressive current conditions. Remarkably, when the current density was reset to 0.1 A g^−1^, NC-2 recovered 223.6 mAh g^−1^, outperforming benchmark materials by >40%, which underscores its structural resilience and rapid Na⁺ diffusion kinetics. This exceptional performance stems from the synergistic interplay of nitrogen doping, defect engineering, and hierarchical porosity, which collectively shorten ionic diffusion pathways and amplify active site accessibility. The GCD profiles (Figure 3f) further corroborated these findings: distinct low-voltage plateaus (<0.2 V) at mild rates (0.1–5.0 A g^−1^) confirmed reversible Na⁺ intercalation into expanded carbon interlayers, while smooth voltage polarization curves implied negligible kinetic limitations and high reaction reversibility.

A complementary EIS analysis (Figure 3g and Figure S6) revealed that NC-2 exhibited a substantially lower charge-transfer resistance (56.9 Ω) compared to HC (98.3 Ω) and other NC variants, aligning with its superior rate capability. Long-term cycling tests at 1.0 A g^−1^ (Figure 3h and Figure S7) demonstrated outstanding stability, with NC-2 retaining 95% of its initial capacity (124.0 mAh g^−1^) after 500 cycles, —significantly exceeding NC-1 (97.1 mAh g^−1^), NC-3 (93.5 mAh g^−1^), and HC (81.0 mAh g^−1^). Near-unity Coulombic efficiency (>99.8%) throughout cycling highlights the stability of the nitrogen-enhanced electrode–-electrolyte interface. The hierarchical sodium storage mechanism involves three stages: (1) surface adsorption at defects/heteroatoms, (2) pore-filling within mesopores, and (3) interlayer insertion, as evidenced by the ex situ structural analysis. During charging, Na⁺ preferentially occupies high-affinity defect sites before penetrating deeper into the carbon matrix, while discharging reverses this sequence, ensuring minimal structural strain and sustained ionic accessibility.

### 2.3. Elucidating Sodium Storage Mechanisms Through Kinetic Analysis

**Figure 4:**
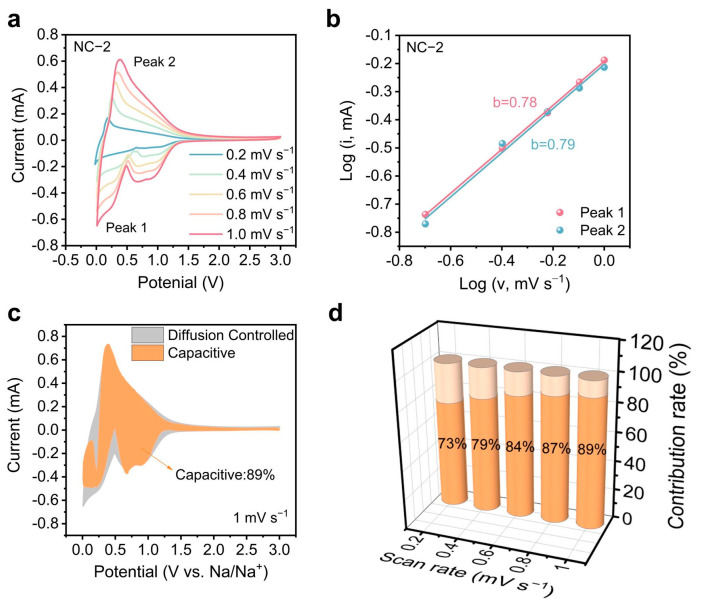
(**a**) CV curves of NC-2 at varying sweep rates ranging from 0.2 mV s^−1^ to 1 mV s^−1^. (**b**) The linear relationship between the peak current and the square root of the sweep rate (*b*-value) for NC-2. (**c**) Capacitance contribution analysis of NC-2 at a sweep rate of 1 mV s^−1^. (**d**) Percentage of pseudocapacitance contribution of NC-2 at different sweep rates.

To deeply explore the kinetic properties of the NC materials, cyclic voltammetry (CV) tests were conducted at different potential sweep rates ranging from 0.2 mV s^−1^ to 1.0 mV s^−1^ (Figure 4a). The CV curves exhibited distinct redox peaks and maintained a similar peak shape as the sweep rate increased. The peak redox current (*I*) and the scan rate (*v*) follow a certain mathematical relationship.

A quantitative kinetic analysis was conducted using the power-law relationship between peak current (*I*) and scan rate (*v*):

*I* (*v*) = *a v^b^* (1)

*log I* = *log a* + *b log v* (2)

where *b*-values approaching 0.5 and 1.0 signify diffusion-controlled and surface-dominated capacitive processes, respectively. Linear fitting of the anodic/cathodic peaks (Figure 4b) yields *b*-values of 0.78 and 0.79, respectively, unambiguously demonstrating that Na^+^ storage in NC-2 is governed by pseudocapacitive charge transfer rather than solid-state diffusion limitations. This behavior contrasts sharply with conventional graphitic carbons (*b* ≈ 0.5) and underscores the critical role of structural defects and nanoscale ordering in enabling surface-driven kinetics.

To deconvolute the exact capacitive contributions, current profiles were further analyzed using the Dunn method:

*I* (*v*) = *k*_1_*v* + *k*_2_*v*^0.5^ (3)

*I* (*v*)/*v*^0.5^ = *k*_1_*v*^0.5^ + *k*_2_ (4)

where a and b are empirical constants, and *k*_1_*v* and *k*_2_*v*^0.5^ represent capacitive and diffusion-controlled currents, respectively. As shown in Figure 4c, the pseudocapacitance contribution of NC-2 is 89% at a scan rate of 1.0 mV s^−1^. At a high current density (1.0 mV s^−1^), the charge storage is, to some extent, necessarily related to the capacitance control. Figure 4d shows the pseudocapacitance contribution ratio of NC-2. With the increase in CV scan rate, the capacitance contribution increases to 73%, 79%, 84%, 87%, and 89%, respectively. The results indicate that nitrogen doping may enhance the adsorption and storage capacity of the material for sodium ions by introducing defects and altering the pore structure.

## 3. Conclusions

We demonstrated the successful preparation of nitrogen-doped hard carbon material (NC-2) with the optimal urea content via the hydrothermal carbonization strategy. During this process, under high temperature and pressure, CS undergoes polycondensation and cross-linking reactions to construct a carbon skeleton. Meanwhile, nitrogen atoms exist in the forms of pyridine nitrogen, graphite nitrogen, and pyrrole nitrogen and are combined with the carbon framework to enhance adsorption and closed-pore storage capacity, strengthen electron/ion transport, and prevent structural collapse and SEI rupture. The subsequent carbonization process further removes noncarbon elements and fixes nitrogen atoms, forming a stable nitrogen-doped hard carbon structure. Nitrogen doping in the carbon matrix introduces defects and active sites, expands the interlayer spacing, and achieves excellent electrochemical performance. These properties collectively enable NC-2 to achieve a high reversible capacity (259 mAh g^−1^ at 0.1 A g^−1^), remarkable rate performance (124.0 mAh g^−1^ at 1.0 A g^−1^), and long-term cycling performance (maintaining a 95% capacity retention rate after 500 cycles at 1 A g^−1^). The proposed synergistic nitrogen doping and defect engineering strategies in this work, combined with a sustainable hydrothermal carbonization process, have great potential for developing next-generation sodium-ion batteries with high performance, low cost, and environmental friendliness.

## Data Availability

The original contributions presented in this study are included in the article/Supplementary Materials. Further inquiries can be directed to the corresponding authors.

## Supplementary Materials

The following supporting information can be downloaded at: https://www.mdpi.com/article/10.3390/ma18102397/s1, Figure S1: (a) XRD spectrum of NC, (b) Raman spectrum of NC. Figure S2: O 1s spectrogram of NC-2 and HC. Figure S3. CV curves of NC-2 and HC at a scan rate of 0.1 mV s^−1^. Figure S4: Charge and discharge curve of (a) NC-1, (b) NC-3. Figure S5: Rate performance of NC. Figure S6: EIS of NC. Figure S7: Cycle performance of NC at 1.0 A g^−1^. Table S1: Electrochemical impedance parameters of NC and HC.
