# Supplementary material for: Synergistic Nitrogen-Doping and Defect Engineering in Hard Carbon: Unlocking Ultrahigh Rate Capability and Long-Cycling Stability for Sodium-Ion Battery Anodes
Source: Materials (Basel). 2025 May 21;18(10):2397. doi: 10.3390/ma18102397 (PMC12113528; doi:10.3390/ma18102397)
Supplement: Supplementary file 1 [file materials-18-02397-s001.zip › materials-3638335-supplementary.pdf]

Synergistic Nitrogen-Doping and Defect Engineering in Hard Carbon: Unlocking Ultrahigh Rate Capability and Long-Cycling Stability for Sodium-Ion Battery Anodes

Na Li [1], Hongpeng Li [2,3,*], Haibo Huang [1,*]

[1] Key Laboratory of Bio-Based Material Science and Technology (Ministry of Education), Northeast Forestry University, Harbin 150040, China
[2] School of Automotive Engineering, Nantong Institute of Technology, Nantong 226001, China
[3] College of Mechanical Engineering, Yangzhou University, Yangzhou 225127, China

* Correspondence: lihongpeng@yzu.edu.cn (H.L.); supernova_bo@nefu.edu.cn (H.H.)

Experimental section

Materials

Corn stalks (CS) were sourced from Hebei Province, China. The CS were thoroughly cleaned, dried, and subsequently ground into a fine powder. Urea (analytical grade) produced by Aladdin Biochemical Technology Co., Ltd. was used. All chemicals were used as received without further purification. Deionized water was utilized throughout the experiments.

Synthesis of Nitrogen-doped Hard Carbon (NC)

Using corn stalks (CS) as the carbon source and urea as the nitrogen source, different concentrations of urea solutions were prepared and mixed evenly. Subsequently, the mixed samples were added into 100 ml high-pressure reactors respectively and subjected to hydrothermal treatment at 180 °C for 12 h. The obtained brown solutions were then washed and freeze-dried. Finally, the precursors were carbonized at 800 °C for 2 h to form the nitrogen-doped hard carbon materials required for the experiment.

The samples were designated as NC-1, NC-2, and NC-3, respectively. The samples that were directly calcined at 800°C for 2 h under a carbon sulfide (CS) atmosphere were named HC.

Characterizations

The microstructural, compositional, and electrochemical properties of the samples were systematically characterized using advanced analytical techniques. Crystallographic structures were analyzed using an X'Pert Powder diffractometer (PANalytical) with Cu Kα radiation (λ = 1.5406 Å). Measurements were performed over a 2θ range of 5° to 80° at a scanning rate of 15° min^{-1}. Surface morphology and microstructure were examined using a field-emission scanning electron microscopy (SEM) (JSM-7500F, JEOL) operated at an accelerating voltage of 5 kV. High-resolution imaging and structural analysis were conducted using transmission electron microscopy (TEM) (JEM-2100, JEOL) at an accelerating voltage of 200 kV. Chemical states and elemental composition were analyzed using an X-ray Photoelectron Spectroscopy (XPS) spectrometer (Thermo Scientific K-Alpha) with Al Kα radiation (1486.6 eV). Binding energies were calibrated using the C 1s peak at 284.8 eV. Graphitization and structural defects were evaluated using a Raman spectrometer (inVia, Renishaw) with a 532 nm laser. Spectra were collected from 500 to 3000 cm^{-1}, and the intensity ratio of the D-band (~1350 cm^{-1}) to the G-band (~1580 cm^{-1}) was calculated to quantify defect density. Gas Sorption Analysis: Specific surface area and pore size distribution were determined using a gas sorption analyzer (BELSORP-Mini II, MicrotracBEL). N_2 adsorption-desorption isotherms were measured at 77 K, and the Brunauer-Emmett-Teller (BET) method was used to calculate the specific surface area. Pore size distributions were derived using the Barrett-Joyner-Halenda (BJH) model.

Electrochemical measurements for Sodium-ion Batteries (SIBs)

The working electrodes were prepared by homogeneously mixing the active material, Super P conductive carbon, and polyvinylidene fluoride (PVDF) binder in a mass ratio of 8:1:1 using N-methyl-2-pyrrolidone (NMP) as the solvent. The resulting slurry was uniformly coated onto copper foil current collectors and vacuum-dried at 80 °C for 12 h to remove residual solvents, yielding electrodes with controlled mass loadings of 1.0-1.5 mg cm^{-2}. CR2032 coin cells were assembled in an argon-filled glovebox (O_2 < 0.01 ppm, H_2O < 0.01 ppm) using sodium metal as the counter electrode, glass fiber separator (Whatman GF/D), and an electrolyte comprising 1 M sodium hexafluorophosphate ($NaPF_6$) in ethylene carbonate/diethyl carbonate (EC/DEC, 1:1 v/v).

Galvanostatic charge/discharge tests were performed on a LAND battery test system (CT3002A) within a voltage window of 0.01-3.0 V (vs. Na^+/Na) at current densities ranging from 0.1 to 5 A g^{-1}. Cyclic voltammetry (CV) and electrochemical impedance spectroscopy (EIS) were conducted using a CHI electrochemical workstation (CS2350H), with CV scans

spanning 0.01-3.0 V at 0.1-1.0 mV s^{-1} and EIS measurements recorded over a frequency range of 10^{-2} to 10^{5} Hz under open-circuit conditions. All electrochemical data were collected at ambient temperature to ensure consistency.

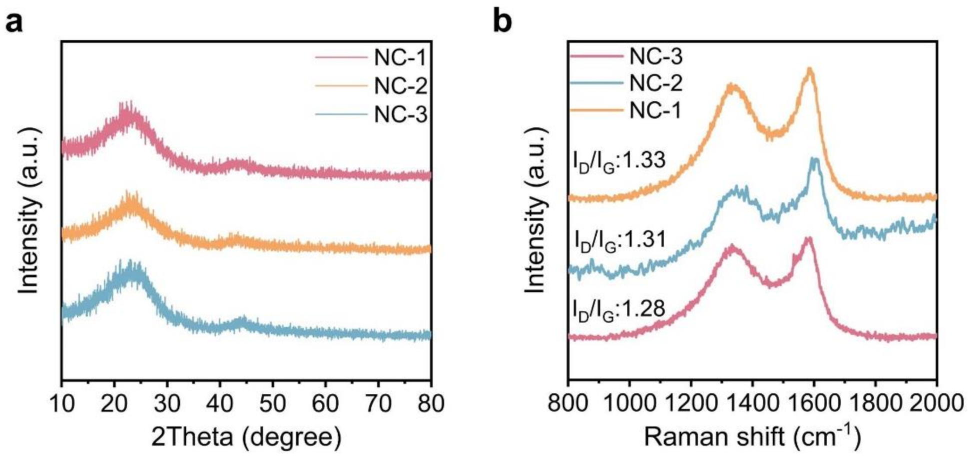

Figure S1. (a) XRD spectrum of NC, (b) Raman spectrum of NC.

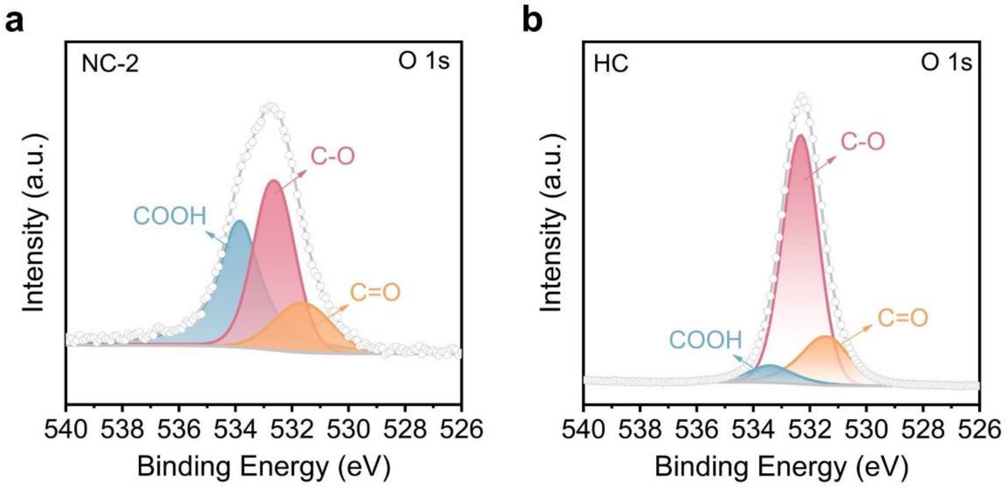

Figure S2. O 1s spectrogram of NC-2 and HC.

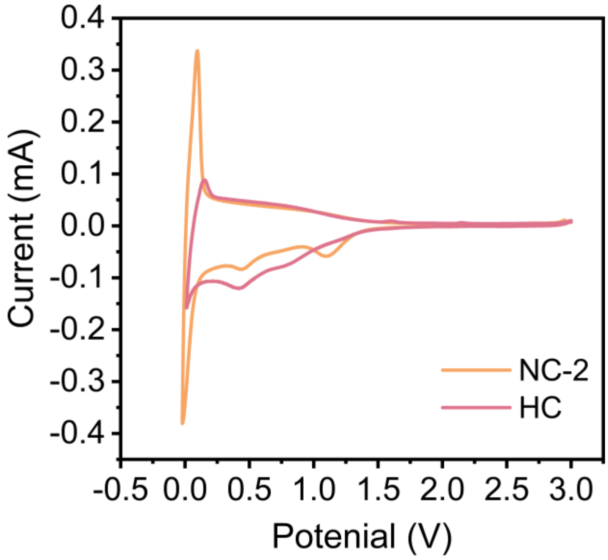

Figure S3. CV curves of NC-2, and HC at a scan rate of 0.1 mV s^{-1}.

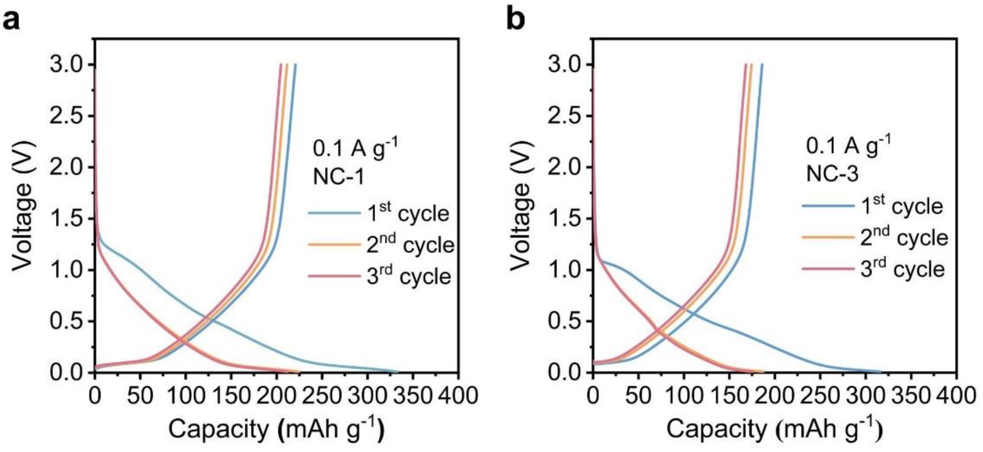

Figure S4. Charge and discharge curve of (a) NC-1, (b) NC-3.

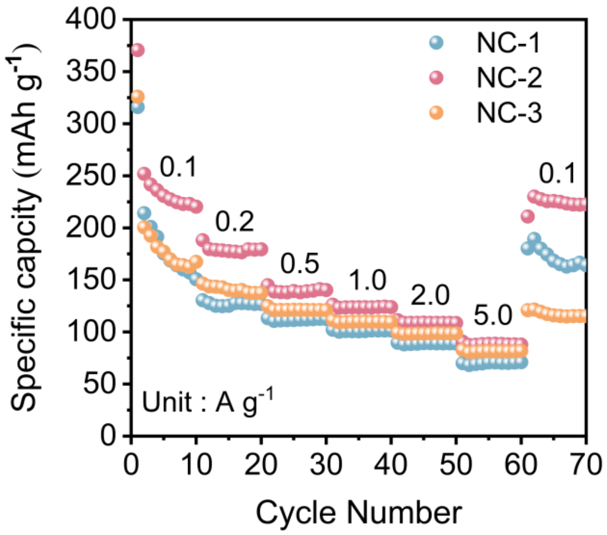

Figure S5. Rate performance of NC.

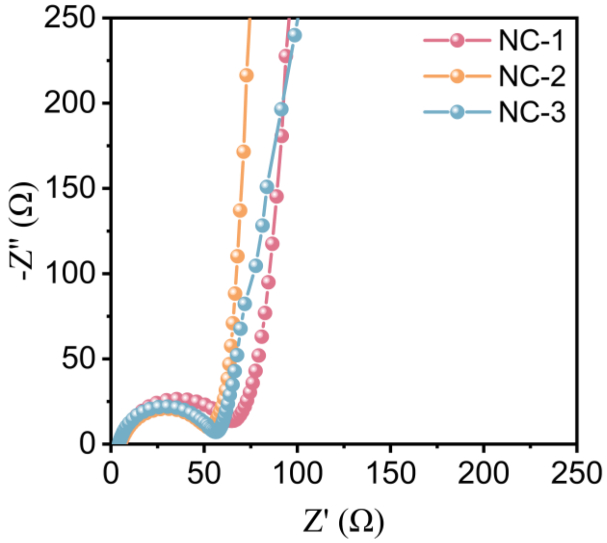

Figure S6. EIS of NC.

Table S1. Electrochemical impedance parameters of NC and HC.

	**NC-1**	**NC-2**	**NC-3**	**HC**
R_{ct} (Ω)	68.6	56.9	59.2	484.8

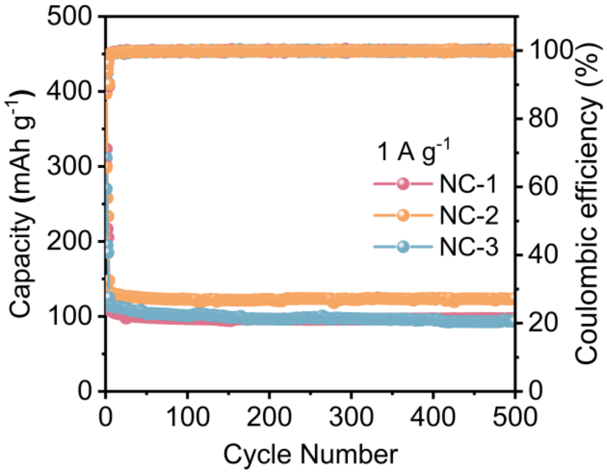

Figure S7. Cycle performance of NC at 1.0 A g^{-1}.
